# Supplementary material for: Management and Survival of Elderly and Very Elderly Patients with Ovarian Cancer: An Age-Stratified Study of 1123 Women from the FRANCOGYN Group
Source: J Clin Med. 2020 May 13;9(5):1451. doi: 10.3390/jcm9051451 (PMC7290352; doi:10.3390/jcm9051451)
Supplement: Supplementary file 1 [file jcm-09-01451-s001.pdf]

Journal of Clinical Medicine

MDPI

Supplementary data

Table S1. Perioperative Complications.

	Population		Age < 65		Age 65 to 74		Age ≥ 75		
	n =	**979**	*n* =	**615**	*n* =	**225**	*n* =	**139**	***p* Value**
		**100%**		**62.8%**		**22.6%**		**14.2%**	
**Peroperative Complications**	181	18.5%	105	17.1%	46	20.4%	30	21.6%	0.321
Hemorrhage/ Transfusion	87	8.9%	47	7.6%	22	9.8%	18	12.9%	
Digestive wound	11	1.1%	8	1.3%	0	0.0%	3	2.2%	
Vascular wound	6	0.6%	4	0.7%	1	0.4%	1	0.7%	
Diaphragmatic wound/Pneumothorax	9	0.9%	8	1.3%	1	0.4%	0	0.0%	
Ureteral or bladder wound	5	0.5%	2	0.3%	1	0.4%	2	1.4%	
Tumor rupture	12	1.2%	8	1.3%	2	0.9%	2	1.4%	
**Postoperative Complications**	202	20.6%	131	21.3%	47	20.9%	24	17.3%	0.566
Hemorrhage/ Transfusion	18	1.8%	9	1.5%	2	0.9%	7	5.0%	
Digestive (fistula, peritonitis, occlusion, pancreatitis)	53	5.4%	36	5.9%	11	4.9%	6	4.3%	
Urinary	19	1.9%	11	1.8%	5	2.2%	3	2.2%	
Lymphocele	19	1.9%	13	2.1%	6	2.7%	0	0.0%	
Abdominal wall (disunion, abscess, evisceration)	33	3.4%	16	2.6%	12	5.3%	5	3.6%	
Respiratory	39	4.0%	30	4.9%	5	2.2%	4	2.9%	
Pneumonia	10	1.0%	6	1.0%	1	0.4%	3	2.2%	
Pneumothorax	4	0.4%	2	0.3%	2	0.9%	0	0.0%	
Pulmonary embolism	11	1.1%	9	1.5%	2	0.9%	0	0.0%	
Pleural effusion	14	1.4%	13	2.1%	0	0.0%	1	0.7%	
Thrombosis	11	1.1%	6	1.0%	5	2.2%	0	0.0%	
Infection (sepsis, pelvic abscess)	22	2.2%	16	2.6%	4	1.8%	2	1.4%	
Neurological deficit	7	0.7%	5	0.8%	1	0.4%	1	0.7%	
**Repeat surgery**	51	5.2%	34	5.5%	12	5.3%	5	3.6%	0.649
**Overall Complications**									
No	428	56%	259	54%	104	58%	65	61%	0.379
Minor complications	263	34%	178	37%	53	30%	32	30%	
Major complications	73	10%	41	9%	22	12%	10	9%	
